# Doctor of pharmacy as a career option: a cross-sectional study exploring PharmD students and practitioners expectations in Jordan

**DOI:** 10.1186/s12960-023-00836-2

**Published:** 2023-06-21

**Authors:** Suha A. AlMuhaissen, Eman Elayeh, Rawia Sharaydih, Taibah M. Abdullah, Aseel AlShalalfeh, Hatim S. AlKhatib

**Affiliations:** 1 Department of Pharmaceutics and Pharmaceutical Technology, School of Pharmacy, The University of Jordan, Amman, 11942 Jordan; 2 Department of Biopharmaceutics and Clinical Pharmacy, School of Pharmacy, The University of Jordan, Amman, 11942 Jordan

**Keywords:** PharmD, Doctor of Pharmacy, Expectations, Professional practice, Job preference, Role in health care

## Abstract

**Objective:**

This study aimed to (1) investigate the expectations and preferences of PharmD students and practitioners regarding their role in the health care system, and (2) to contrast those expectations and preferences of PharmD practitioners with real-life practice in Jordan.

**Methods:**

Two cross-sectional descriptive questionnaires were used to collect data from PharmD students and PharmD practitioners in Jordan. A total number of 330 students and 280 practitioners were interviewed. The responses to all questions were encoded, entered, and summarized as frequencies and percentages or as means and standard deviations. Comparisons between groups were performed using Chi-square test. A *p*-value of < 0.05 was considered significant.

**Results:**

Both PharmD students and practitioners chose working as a clinical pharmacist in a hospital as their first-choice job. However, their second and third jobs choices were significantly different as practitioners opted for income as a main criterion for job selection. Interestingly, salary expectations by PharmD students were significantly higher than the reality as reported by PharmD practitioners. Both students and practitioners placed the work environment as the highest priority criterion for making a work choice on the work environment. In general, both students and practitioners agree on the ideal roles of PharmD graduate with the issues of prescribing and compounding responsibilities being the ones with the highest disparity between practitioners and students. Significant differences were found between the student’s and practitioner’s perceptions of the ideal role of a PharmD in and the current professional practice in Jordan.

**Conclusion:**

Job preferences and salary expectations differ significantly between students and practitioners. Professional orientation of PharmD. students should be implemented to minimize misconceptions of their job nature, availability, and compensations. The fact that students do not prefer to work in a community pharmacy should be addressed by educational institutions and professional organizations. The prescribing and compounding responsibilities of pharmacists should be also emphasized in the curricula of pharmacy schools and worked in by professional organization to achieve optimal implementation in real-life practice.

## Introduction

Clinical pharmacy is a specialized field of pharmacy that focuses on optimizing medication-related outcomes for patients throughout the medication management process [1]. Recent studies have reported that the implementation of clinical pharmacy services can minimize medication errors, reduce hospital costs, and improve pharmacotherapeutic outcomes for patients [2]. For example, clinical pharmacy services were reported to prevent 64.19% of medical errors in a group of hospitalized patients with diabetes, moreover, the same study found that the inclusion of clinical pharmacists in hospital rounds reduced the number of drug interactions in intensive care patients by 65% [3].

In 2014, the American College of Clinical Pharmacy (ACCP) set forth ACCP’s expectations for clinical pharmacists’ roles and defined the components of the clinical pharmacist's process of care including: assessment of the patients, evaluation of medication therapy, development and implementation of a plan of care, and follow up, evaluation and medication monitoring [4].

Jordan, a middle eastern Arab country with a population of about 10 million, is classified by the World Bank as an upper middle-income economy [5]. Jordan’s health care services are internationally renowned, and Jordan is a leading destination for medical tourism that generates over USD 1 billion by attracting 250 000 patients from neighboring countries every year [6]. Additionally, Jordan has been ranked as the number one medical tourism destination in the region and fifth worldwide by the World Bank organization [6, 7]. The proportion of spending on medicines in Jordan was about one-fourth of the health care spending and constituted 2% of GDP which is a high value for a middle-income country considering that, for the same year, expenditures on medicines in the European Union represented 1.6% of GDP [6].

In support of such a dynamic health care sector, health care professionals in Jordan are trained by a modern higher education system [6].

There are currently 18 pharmacy schools in Jordan, five of which are public schools, while the other 13 are private. All pharmacy schools in Jordan offer a professional Bachelor of Science in Pharmacy programs (BSc Pharm) while only two public schools offer an additional Doctor of Pharmacy program (PharmD). Jordan University of Science and Technology (JUST) started enrolling students in the PharmD program in the year 2000 and The University of Jordan (UJ) followed shortly after in 2005. The 2018 National Human Resources for Health Observatory Annual Report shows that that (13 443) students were enrolled in eighteen pharmacy schools with 60% of them being Jordanian nationals and 40% being non-Jordanians [8].

The PharmD programs offered by JUST and UJ are professional doctorate programs qualifying graduates to become pharmacists, PharmD programs in Jordan are different from an academic doctorate, such as Doctor of Philosophy (PhD), that focuses on knowledge or original research production [9].

PharmD programs in Jordan are coursework-based degrees with students enrolling after completing high school. To meet graduation requirements, students are required to complete 216 (UJ) or 210 (JUST) credit hours in 6 academic years. An important component of the programs is the experiential practical training where structured field and simulated training are carried out under faculty members’ supervision in community and hospital pharmacies. The experiential training culminates in a series of clinical rotations, known as the clerkships, that span the final sixth year of the program. Each internship course includes about 40 h per week of on-site experience (Experiential Programs) where students get exposure to ward rounds as part of the multidisciplinary team, run quality improvements plans (QIP's) and present actual case-studies while being supervised by practitioner faculty members [10, 11].

PharmD curricula in Jordan aim to provide students with basic knowledge in pharmaceutical and biomedical sciences, train them in professional pharmacy services and allow them to develop attitudes needed for practice.

The professional competencies of PharmD programs in Jordan were developed, by a committee that included different academic and professional stake holders, to define the desired knowledge, skills, and behaviors of graduates of PharmD programs. These competencies were then adopted officially by the Jordanian Accreditation and Quality Assurance Commission for Higher Education Institutions in 2020.

The professional competencies of PharmD programs in Jordan are classified in seven categories covering different aspects of professional pharmacy practice and the potential roles of graduates in the national health system. These categories include (i) dispensing of medicines; (ii) patient care; (iii) pharmaceutical compounding; (iv) pharmaceutical supply and marketing; (v) regulatory affairs; (vi) interpersonal and communication skills; and (vii) continuous professional development [12].

The PharmD programs in Jordan are comparable to other programs around the world (U.S., Canada, Hungary, Italy, Japan, South Korea, Pakistan, Saudi Arabia, Thailand, Benin, Cameroon, Republic of Congo, Senegal, Tunisia, Nigeria and Gana) in terms of program duration and focus on pharmaceutical care and the clinical role of pharmacists in a health care team [9].

PharmD graduates from UJ and JUST can practice as pharmacists in Jordan upon licensing by the Ministry of Health and registration with the Jordan Pharmacists Association. The licensing and registration require, besides having a pharmacy degree, the completion of 1440 h of professional training. Graduates from non-Jordanian universities are required also to pass a pharmacy license examination. In addition, starting in 2023, completion of an annual continuing professional development program will become a requirement for maintaining registration with the Jordan Pharmacists Association and licensing by the Ministry of Health [13].

Few studies have examined PharmD students’ expectations of their future roles as clinical pharmacists in comparison with real-life practices in different countries. In the USA, PharmD students have indicated that their education better prepared them for their expected career than did BSc Pharm students, they also had a more positive outlook regarding future career opportunities [14]. Another study reported that controllable work schedule and career fulfillment had the most influence on PharmD student’s career choices, in addition, those students viewed hospital practice as the optimal career environment [15].

A study conducted in 13 Middle Eastern, Arabic speaking countries showed that most of BSc Pharm graduates work in community pharmacy, followed by hospitals, industry, and others (e.g., regulatory agencies and academia). Most pharmacists in Jordan work in the private sector work as community pharmacists, institutional positions are also available, as are opportunities in the thriving pharmaceutical industry, only Jordanians are eligible for licensure and practice within the country [16].

One study [17] has attempted to investigate students' career choices at the University of Jordan School of Pharmacy, the results showed that the majority of PharmD students lacked knowledge about their degree, and awareness workshops were strongly recommended by authors.

This study aimed to (1) investigate the expectations of PharmD students and practitioners of their role in the health care system, and (2) contrast the expectations of PharmD practitioners of their professional roles with real-life practice in Jordan.

## Methods

### Data collection

Data for this project were collected using two cross-sectional descriptive questionnaires. One questionnaire was used for data collection from PharmD students (PS) in the two Jordanian universities that offer a PharmD degree (JUST and UJ). The second questionnaire was used to collect data from PharmD practitioners (PP) in Jordan. The data collection tool was built by authors (SA and EE) based on information collected from an extensive review of published relevant literature [17–20] and the authors own observations and experience with the practice.

The students’ questionnaire was divided into two sections collecting data on: (i) students’ demographics and (ii) their job preferences and expected/ideal role of PharmD graduates within the health care team.

The practitioners’ questionnaire was designed in three sections collecting data on: (i) practitioner’s demographics; (ii) their job preferences and expected/ideal role of PharmD graduates within the health care team; and (iii) the real-life role played by those PharmD practitioners.

The questionnaires were designed to answer the following research questions:

- What are the career goals of PS and PP and are there any significant differences in this regard between the two groups? (*p* = *0.05*)
- What aspects of an ideal job and its conditions are most important and are there any significant differences in this regard between PS and PP? (*p* = *0.05*)
- What is the perceived ideal role of PharmD graduate within the health care team and are there any significant differences in this regard between PS and PP? (*p* = *0.05*)

Furthermore, the practitioners’ questionnaire aimed to answer an additional question:

- Are there any differences between the expected (perceived or ideal) role of a PharmD graduate and the real-life practice in Jordan?

The two data collection tools were assessed for validity and reliability. Initial assessment of the two questionnaires was done by a practicing PharmD graduate and a senior year PharmD student. The pilot study included 25 students and 25 practitioners who were randomly selected. The two questionnaires were, accordingly, modified before the main data collection started. Further statistical validation of the questionnaires’ reliability was assured by calculating Cronbach’ Alpha value which was found to be 0.752 for students’ questionnaire and 0.876 for practitioners’ questionnaire indicating consistency of the two tools.

The main data collection took place between December 2019 and February 2020. A total number of 330 students and 280 practitioners were interviewed.

There were no exclusion criteria as participants were randomly chosen regardless of their age, gender, ethnicity, geographic location of site of practice and place of study. Data collection was carried out by three of the authors who contacted participants in person or by phone calls, introduced themselves, took the participant's oral consent before the starting a structured interview process.

Sample size calculation was made using Raosoft™ sample size calculator with 95% confidence level. A sample size of 270–385 respondents for each group (students and practitioners) was determined to meet the required confidence level. The total number of Pharm D students at the time of study was obtained from the records of the registration departments of The University of Jordan and the Jordan University of Science and Technology, the only two universities offering a Pharm D program in Jordan, while the total number of practitioners was obtained from the Jordan Pharmacists Associations.

To minimize social desirability bias, assurance was given to interviewees that discussions would be confidential, and their responses would be anonymized. Collected data were stored with the corresponding author and further analysis was done anonymously.

### Statistical analysis

The responses to all questions were encoded, entered, and analyzed using SPSS® 23.0 (IBM, Armonk, NY). Responses were then summarized as frequencies and percentages for categorical variables, and as means and standard deviations (or medians and interquartile ranges) for continuous variables.

Comparisons between groups were performed using Chi-square test. A *p*-value of < 0.05 was considered significant. All hypothesis testing was two-sided.

## Results

In total, 330 PS and 280 PP have completed the questionnaire, with 100% and 97%, response rates, respectively.

### Career goals of PharmD students and practitioners

The vast majority of both, PS (82.1%) and PP (82.5%), were females. As expected, almost all PP (98.2%) and only 29.7% of PS had prior training in hospitals. More than 99% of all PP respondents are graduate of Jordanian universities (Table 1).

**Table 1:** Demographics and characteristics of participants

	Students, *N* = 330 *N* (%)	Practitioners, *N* = 280 *N* (%)
Gender		
Female	271 (82.1)	231 (82.5)
Male	59 (17.9)	49 (17.5)
Prior work experience or training in hospital		
Yes	98 (29.7)	275 (98.2)
No	232 (70.3)	5 (1.8)
Country of graduation		
Jordan	328 (99.4)	279 (99.6)
The University of Jordan	213 (64.5)	
Jordan University for Science and Technology	115 (34.9)	
Others	2 (0.6)	1 (0.4)
Marital status		
Single	318 (96.4)	187 (66.7)
Married and others	12 (3.6)	93 (33.3)
Current year status		
Second year	43 (13)	NA
Third year	71 (21.5)	NA
Fourth year	92 (27.9)	NA
Fifth year	54 (16.4)	NA
Sixth year	70 (21.2)	NA
Year of graduation/ work experience, years (median (IQR))	NA	1(0.5–4)
GPA (mean ± SD)	3.2 ± 0.44	NA
Age (mean ± SD)	22.1 ± 1.3	26.7 ± 2.6

*GPA* grade point average

*NA* not applicable

Table 2 illustrates job preferences as stated by PS and PP. The highest percentages (82.4% of PS and 45% of PP) prefer working as a clinical pharmacist in a hospital, yet the PSs’ preference is significantly higher (*p* < *0.001*). The PS second and third ranking preferences are “university teacher” (32.4%) and “researcher” (26.1%), respectively. Working as a medical representative (14.6%) and in a hospital pharmacy (13.6%) are the second and third top ranking job preferences by PP. Interestingly job preferences are significantly different between students and practitioners. In the same trend, salary expectations by PS are significantly higher (*p* < *0.001*) than the reality as reported by PP.

**Table 2:** Place of work—job preferences and salary; expectations and reality

	Students, *N* = 330 *N* (%)	Practitioners, *N* = 280 *N* (%)	Chi-square
Place of work/ preferences^@^			
Community pharmacy (chain)	55 (16.7)	37 (13.2)	*F* = 1.410, *p* = *0.141*
Community pharmacy (Independent)	56 (17)	28 (10)	*F* = 6.197, *p* = 0.008*
Medical representative	61 (18.5)	41 (14.6)	*F* = 1.601, *p* = *0.123*
Clinical pharmacist in a hospital	272 (82.4)	42 (45)	*F* = 275.685, < 0.001*
Hospital pharmacy	64 (19.4)	38 (13.6)	*F* = 3.688, *p* = 0.034*
Pharmaceutical industry	59 (17.9)	7 (2.5)	*F* = 37.128, *p* < 0.001*
University teacher or professor	107 (32.4)	29 (10.4)	*F* = 42.577, < 0.001*
Researcher	86 (26.1)	17 (6.1)	*F* = 43.127, *p* < 0.001*
Regulatory affairs and drug registration	24 (7.3)	8 (2.9)	*F* = 5.942, *p* = 0.011*
Policy makers	4 (1.2)	1 (0.4)	*F* = 1.362, *p* = *0.242*
Regulatory Agency (JFDA)	36 (10.9)	6 (2.1)	*F* = 18.156, *p* < 0.001*
Work outside Jordan	118 (35.8)	102(36.4)	*F* = 0.030, *p* = *0.465*
No intent to work	2(0.6)	None	*F* = 1.703, *p* = *0.292*
Others (e.g., health insurance companies, self-employed, online blogs/translations)	161 (48.8)	43 (15.4)	*F* = 8.348, *p* = 0.003*
Salary consideration	Expectations	Reality	
≤ 400 JD	23 (7)	62 (22.2)	
400–600 JD	140 (42.4)	130 (46.6)	
600–800 JD	114 (34.5)	44 (15.8)	
≥ 800 JD	53 (16.1)	43 (15.4)	*F* = 46.373, *p* < 0.001*

^@^valid percent (values don’t sum up to 100%)

### Factors that affect career choice of PharmD students and practitioners

Table 3 summarizes the factors affecting job selection by both PS and PP. Both, PS (40.6%) and PP (38.9%), selected the work environment as the most important factor affecting their job choice. More than one-third of PS (34.2%) are concerned with salary and benefits like bonus package and health insurance, while PP (25%) give a higher importance to the flexibility of the work schedule and the number of days off. Generally, factors upon which PharmD graduates build their job choices are highly variable.

**Table 3:** Job selection consideration—students and practitioners – *N* (%)

Factor	Very low importance	Low importance	Neutral	High importance	Very high importance	Chi-square
Location of the work						
Students	11 (3.3)	29 (8.8)	123 (37.3)	99 (30)	68 (20.6)	*F* = 15.311
Practitioners	9 (3.2)	32 (11.4)	75 (26.8)	117 (41.8)	1 (0.4)	*p* = *0.018*
Work environment						
Students	3 (0.9)	10 (3)	50 (15.2)	133 (40.3)	134 (40.6)	*F* = 5.529
Practitioners	8 (2.9)	15 (5.4)	42 (15)	106 (37.9)	109 (38.9)	*p* = *0.237*
Salary and benefit (bonus package, health insurance, …)						
Students	None	6 (1.8)	69 (20.9)	142 (43)	113 (34.2)	*F* = 32.283
Practitioners	13 (4.6)	24 (8.6)	46 916.4)	115 (41.1)	82 (29.3)	*p* < *0.001*
Flexibility of the work schedule						
Students	10 (3)	18 (5.5)	113 (34.2)	130 (39.4)	59 (17.9)	*F* = 16.102
Practitioners	15(5.4)	24(8.6)	64 (22.9)	107 (38.2)	70 (25)	*p* = *0.007*
Number of work hours per week						
Students	3 (0.9)	27 (8.2)	136 (41.2)	113 (34.2)	51 (15.5)	*F* = 14.154
Practitioners	13(4.6)	30(10.7)	74(26.4)	104(37.1)	59(21.1)	*p* = *0.007*
Level of responsibility						
Students	7 (2.1)	12 (3.6)	75 (22.7)	143 (43.3)	93 (28.2)	*F* = 10.2279
Practitioners	7 (2.5)	28 (10)	61 (21.8)	113(40.4)	71 (25.4)	*p* = *0.036*
Number of holidays per week						
Students	8 (2.4)	20 (6.1)	114 (34.5)	115 (34.8)	73 (22.1)	*F* = 9.705
Practitioners	10 (3.6)	24 (12.1)	81 (28.9)	118 (42.1)	47 (16.8)	*p* = *0.046*

### Ideal vs current role of PharmD graduate from students’ and practitioners’ point of view

Tables 4 and 5 explore the ideal expectations by PS and PP of the different roles of a PharmD graduate in the health care system and the current roles of PharmD graduate as reported by PP from their real-life experience.

**Table 4:** Expected roles of a clinical pharmacist, ideally—students’ and practitioners’ perspectives –, presented as *N* (%)

	Students *N* = 330	Practitioners *N* = 280	All *N* = 610	Chi-square
Medication management and patients’ health care				
1. History taking and reconciliation	294 (89.1)	262 (93.6)	556 (91.1)	*F* = 4.819, *p* = *0.090*
2. Follow guidelines and protocols to ensure that patient receive optimal drug therapy and provide an effective and safe medicine	321 (97.3)	271 (96.8)	592 (97)	*F* = 3.750, *p* = *0.153*
3. Monitor and adjust patient therapy	312 (94.5)	272 (97.1)	584 (95.7)	*F* = 6.156, *p* = *0.046*
4. Detect and resolve drug-related problems (side effects, drug–drug interactions)	318 (96.4)	276 (98.6)	594 (97.4)	*F* = 2.376, *p* = *0.305*
5. Have prescribing responsibility and provide repeat medication independently	162 (49.1)	204 (72.9)	366 (60)	*F* = 36.075, *p* < *0.001*
6. Update clinical record	233 (70.6)	237 (84.6)	470 (77)	*F* = 17.671, *p* < *0.001*
Patient examination and screening				
1. Conduct basic physical assessments	153 (46.4)	165 (58.9)	319 (52.3)	*F* = 11.073, *p* = *0.011*
2. Play an important role in reducing antibiotic resistance by changing antibiotics or dosing based on laboratory values	305 (92.4)	268 (95.7)	573 (93.9)	*F* = 6.584, *p* = *0.037*
3. Order and review laboratory tests	240 (72.3)	230 (82.1)	470 (77)	*F* = 15.109, *p* = *0.001*
4. Conduct drug allergy assessment	274 (83)	243 (86.8)	517 (84.8)	*F* = 1.659, *p* = *0.436*
5. Review immunizations and provide vaccines where required	236 (71.5)	229 (81.8)	466 (76.4)	*F* = 11.710, *p* = *0.008*
Drug information and education				
1. Answer medication-related questions from patients and health care professionals	321 (97.3)	272 (97.1)	593 (97.2)	*F* = 0.042, *p* = *0.979*
2. Counseling and education on medication management (doses, frequency, onset of action, side effects, drug interactions, store condition) and provide evidence-based drug information and recommendations	322 (97.6)	276 (98.6)	598 (98)	*F* = 1.267, *p* = *0.531*
3. Provide non-pharmacological treatment (diet, exercise, smoking)	267 (80.9)	272 (97.1)	539 (88.4)	*F* = 38.837, *p* < *0.001*
4. Carry out compounding procedures to produce an effective and safe medicine (preparation of IV antibiotics, total parenteral nutrition)	221 (67)	228 (81.4)	449 (73.6)	*F* = 16.456, *p* < *0.001*
5. Act as preceptor for students	255 (77.3)	247 (88.2)	502 (82.3)	*F* = 13.458, *p* = *0.001*
6. Education of general physicians and other practice staff and mentor new prescribers	263 (79.7)	232 (82.9)	495 (81.1)	*F* = 1.244, *p* = *0.537*
7. Design prevention, intervention, and educational strategies for individuals and communities to manage chronic disease and improve health and wellness	272 (82.4)	241 (86.1)	513 (84.1)	*F* = 1.897, *p* = *0.387*
8. Educate individuals and communities to manage chronic diseases and improve health and wellness	296 (89.7)	265 (94.6)	561 (92)	*F* = 5.681, *p* = *0.058*
Collaboration and liaison				
1. Regular participation on patient care rounds	301 (91.2)	260 (92.9)	561 (92)	*F* = 0.193, *p* = *0.908*
2. Refer to general physician and other health professional	304 (92.1)	262 (93.6)	566 (92.8)	*F* = 0.507, *p* = *0.776*
3. Participate in multidisciplinary reviews of patients	291 (88.2)	240 (85.7)	531 (87)	*F* = 2.372, *p* = *0.305*
4. Liaison role between hospital and community pharmacists across health sector	248 (75.2)	218 (77.9)	466 (76.4)	*F* = 5.674, *p* = *0.059*
5. Collaboration with other health care professionals (GPs, dietician, nurse practitioners)	316 (95.8)	270 (96.4)	586 (96)	*F* = 1.24, *p* = *0.444*
6. Attend and present at meetings with other healthcare staff and communicate effectively with patients, caregivers, pharmacy personnel, other health care professionals, community members, policy makers and administrators	306 (92.7)	270 (96.4)	576 (94.4)	*F* = 3.984, *p* = *0.136*
7. Interpret results derived experimentally or by simulation - Summarize and present experimentally or simulated derived data - Write a scientifically sound report of an experiment - Utilize IT in data management and presentation	273 (82.7)	230 (82.1)	503 (82.5)	*F* = 1.188, *p* = *0.552*
8. Establish collaborative practice agreements with physician	297 (90)	252 (90)	549 (90)	*F* = 1.673, *p* = *0.433*
Quality assurance				
1. Play an important role in improving patient health outcome	326 (98.8)	271 (96.8)	598 (98)	*F* = 4.667, *p* = *0.198*
2. Physician workload could be minimized	276 (83.6)	240 (85.7)	516 (84.6)	*F* = 1.602, *p* = *0.449*
3. Help to reduce drug costs	258 (78.2)	260 (92.9)	518 (84.9)	*F* = 26.371, *p* < *0.001*
4. Participate in and coordinate research activities	294 (89.1)	262 (93.6)	556 (91.1)	*F* = 4.300, *p* = *0.117*
5. Developing clinical guidelines and prescribing templates	272 (82.4)	240 (85.7)	512 (83.9)	*F* = 1.238, *p* = *0.536*

**Table 5:** Expected roles of a clinical pharmacist, ideal vs current practice—practitioners’ perspectives –, *N* = 280, presented as *N* (%)

	Ideally	Current	Chi-square
Medication management and patients’ health care			
1. History taking and reconciliation	262 (93.6)	151 (53.9)	*F* = 114.649, *p* < *0.001*
2. Follow guidelines and Protocols to ensure that patient receive optimal drug therapy and provide an effective and safe medicine	271 (96.8)	182 (65)	*F* = 90.628, *p* < *0.001*
3. Monitor and adjust patient therapy	272 (97.1)	166 (59.3)	*F* = 116.897, *p* < *0.001*
4. Detect and resolve drug-related problems (side effects, drug–drug interactions)	276 (98.6)	195 (69.6)	*F* = 86.944, *p* < *0.001*
5. Have prescribing responsibility and provide repeat medication independently	204 (72.9)	82 (29.3)	*F* = 108.433, *p* < *0.001*
6. Update clinical record	237 (84.6)	96 (34.3)	*F* = 152.591, *p* < *0.001*
Patient examination and screening			
1. Conduct basic physical assessments	165 (58.9)	82 (29.3)	*F* = 49.995, *p* < *0.001*
2. Play an important role in reducing antibiotic resistance by changing antibiotics or dosing based on laboratory values	268 (95.7)	167 (59.6)	*F* = 104.705, *p* < *0.001*
3. Order and review laboratory tests	230 (82.1)	132 (47.1)	*F* = 75.177, *p* < *0.001*
4. Conduct drug allergy assessment	243 (86.8)	149 (53.2)	*F* = 77.894, *p* < *0.001*
5. Review immunizations and provide vaccines where required	229 (81.8)	89 (31.8)	*F* = 147.925, *p* < *0.001*
Drug information and education			
1. Answer medication-related questions from patients and health care professionals	272 (97.1)	234 (83.6)	*F* = 29.778, *p* < *0.001*
2. Counseling and education on medication management (doses, frequency, onset of action, side effects, drug interactions, store condition) and provide evidence-based drug information’s and recommendations	276 (98.6)	239 (85.4)	*F* = 32.180, *p* < *0.001*
3. Provide non-pharmacological treatment (diet, exercise, smoking)	272 (97.1)	204 (72.9)	*F* = 65.374, *p* < *0.001*
4. Carry out compounding procedures to produce an effective and safe medicine (preparation of IV antibiotics, total parenteral nutrition)	228 (81.4)	92 (32.9)	*F* = 143.362, *p* < *0.001*
5. Act as preceptor for students	247 (88.2)	190 (67.9)	*F* = 39.029, *p* < *0.001*
6. Education of general physicians and other practice staff and mentor new prescribers	232 (82.9)	132 (47.1)	*F* = 83.410, *p* < *0.001*
7. Design prevention, intervention, and educational strategies for individuals and communities to manage chronic disease and improve health and wellness	241 (86.1)	113 (40.4)	*F* = 138.372, *p* < *0.001*
8. Educate individuals and communities to manage chronic diseases and improve health and wellness	265 (94.6)	201 (71.8)	*F* = 51.899, *p* < *0.001*
Collaboration and liaison			
1. Regular participation on patient care rounds	260 (92.9)	98 (35)	*F* = 209.553, *p* < *0.001*
2. Refer to general physician and other health professional	262 (93.6)	208 (74.3)	*F* = 40.093, *p* < *0.001*
3. Participate in multidisciplinary reviews of patients	240 (85.7)	133 (47.5)	*F* = 113.091, *p* < *0.001*
4. Liaison role between hospital and community pharmacists across health sector	218 (77.9)	98 (35)	*F* = 122.517, *p* < *0.001*
5. Collaboration with other health care professionals (GPs, dietician, nurse practitioners)	270 (96.4)	174 (62.1)	*F* = 102.257, *p* < *0.001*
6. Attend and present at meetings with other healthcare staff and communicate effectively with patients, caregivers, pharmacy personnel, other health care professionals, community members, policy makers and administrators	270 (96.4)	143 (51.1)	*F* = 150.614, *p* < *0.001*
7. Interpret results derived experimentally or by simulation - Summarize and present experimentally or simulated derived data - Write a scientifically sound report of an experiment - Utilize IT in data management and presentation	230 (82.1)	120 (42.9)	*F* = 106.608, *p* < *0.001*
8. Establish collaborative practice agreements with physician	252 (90)	112 (40)	*F* = 155.343, *p* < *0.001*
Quality Assurance			
1. Play an important role in improving patient health outcome	271 (96.8)	221 (78.9)	*F* = 44.932, *p* < *0.001*
2. Physician workload could be minimized	240 (85.7)	119 (42.5)	*F* = 114.161, *p* < *0.001*
3. Help to reduce drug costs	260 (92.9)	169 (60.4)	*F* = 82.483, *p* < *0.001*
4. Participate in and coordinate research activities	262 (93.6)	157 (56.1)	*F* = 110.883, *p* < *0.001*
5. Developing clinical guidelines and prescribing templates	240 (85.7)	88 (31.4)	*F* = 180.077, *p* < *0.001*

Five major domains, each covered by 5 – 8 possible missions/roles of PharmD graduates (totally 32 items), were investigated. These missions/roles were originally adapted from the American Association of Colleges of Pharmacy (AACP) white paper on clinical pharmacist competencies (18). The domains include (1) medication management and patients’ health care; (2) patient examination and screening; (3) drug information and education; (4) collaboration and liaison; and (5) quality assurance.

More than two-thirds (ranging between 67% to 98.8%) of PS believe that 30 out of the 32 missions/roles items are ideal missions/roles of PharmD graduates in the health care system. Similar outcomes are seen with PP, where more than two-thirds of PP respondents (72.9% to 98.6%) agree that 31 out of the 32 items should be considered ideal missions/roles of PharmD graduate.

Some significant differences between PS and PP in their expectations of the ideal role of PharmD graduate are noticeable. The major differences appear in two important roles: (1) while almost (80%) of PP believe that PharmD graduate have prescribing responsibility and should be able to provide repeat medication independently, only 49.1% PS agree on that, and (2) a significantly larger proportion of PP (81.4%) in comparison to PS (67%) believe that PharmD graduate should carry out compounding procedures to produce an effective and safe medicine including the preparation of IV antibiotics and total parenteral nutrition.

The most alarming outcomes are seen when comparing the ideal and real-life roles of PharmD graduate in the health care team from PP real-life experience. PP have reported that most of the ideal roles of a PharmD graduate in the health care system are not being implemented in the current practice. An important example on this is the lack of regular participation in patient care rounds which is reported to be practiced by 35% of PP in comparison to 91.2% of PS and 92.9% of PP who agree on it being an important role of a PharmD graduate in the health care system.

Another remarkable example of discrepancy between the ideal and practical roles of PharmD graduates can be found considering their role in designing prevention, intervention, and educational strategies for individuals and communities to manage chronic disease and improve health and wellness. On one hand, 82.4% of PS and 86.1% of PP believe it to be an integral role of a PharmD, on the other hand, only about 40% of PP have seen it being implemented in the current practice.

The presence of a significant difference between what is believed to be an ideal role of PharmD graduate and what is really being practiced regarding all suggested missions of a PharmD graduate is evident.

## Discussion

National Human Resources for Health Observatory Annual Report shows that the total number of Pharmacists in Jordan is 15 400 and the ratio of Pharmacists to 10 000 populations is 14.9. The distribution of pharmacists in Jordanian Governorates varies from 28.7 Pharmacists per 10.000 citizens in Amman to 2.6 Pharmacists per 10.000 citizens in Ajloun [8].

Most pharmacists in Jordan work in community pharmacies, followed by pharmaceutical companies and hospitals. The private sector is the main employer of health workers in Jordan as 91% of pharmacists work in private community pharmacies, private hospitals, pharmaceutical companies, and cosmetics and medical supplies companies [8]. Public sector pharmacists work in the Ministry of Health, university hospitals, Royal Medical Services, and not-for-profit organizations such as the UNRWA [16].

An important point regarding the PharmD graduates career options is that the Jordanian Pharmaceutical Licensing Regulation 2019 defines a clinical pharmacist as pharmacist who is licensed to practice pharmacy by the Ministry of Health and holds either a PharmD or an MSc in Clinical pharmacy degree. The same regulation requires the presence of a clinical pharmacist for each 50 or less hospital beds as a requirement for the licensing of a hospital pharmacy [21]. This provides an almost exclusive professional practice venue for PharmD graduates where they can apply their specialized training in sixth year clinical clerkships.

The main aim of this study was to investigate the expectations of PS and PP regarding their role in the health care system, and to highlight the differences between the expectations of PP about the profession and the real practice in Jordan.

### Career goals of PharmD students and practitioners

More than 80% of both PS and PP in this study were females. The observed skew in the sample toward females has also been noticed in previous studies in Jordan and elsewhere [17, 19, 22]. Based on data provided by the Jordan Pharmacy Syndicate, female pharmacists comprise 69.9% of the total pharmacists, 83.2% of PharmD holders and 69.1% of BSc holders. Data provided by the School of Pharmacy at the University of Jordan show that female students comprise 76.9% of PharmD students and 84.9% of BSc students.

The highest percentage of respondents (82.4% of PS and 45% of PP) prefer working as clinical pharmacists in a hospital, yet the students’ preference is significantly higher (*p* < *0.001*). These findings are consistent with results from several studies from Jordan [17] Malaysia [23], United Kingdome [24] and Saudi Arabia [25]. On the other hand, retail chains and independent community pharmacies were the preferred working areas by PharmD students in USA [26].

Moreover, this study revealed that job preferences change significantly upon graduation and entry to the job market. While students second and third preferred jobs are university teachers or professors (32.4%) and researchers (26.1%), respectively, working as a medical representative (14.6%) and in a hospital pharmacy (13.6%) were the second and third top listed preferred jobs by practitioners. This may be explained by the realization by PP that vacancies for clinical pharmacists in hospitals are limited in comparison to the number of PharmD graduates in addition to the lower-than-expected salaries of PharmD practitioners in hospitals. The limited number of vacancies for hospital pharmacists in hospital should be particularly emphasized as the minimum number of clinical pharmacists required to satisfy the licensing requirements of all hospitals in Jordan based on the total number of hospital beds (about 14 700 hospital bed) is only 296 (8).

The relative low preference by PS for working in a community pharmacy, although it constitutes most of pharmacy job opportunities available in Jordan is noteworthy, it should be further investigated and appropriately addressed. One of the most important tasks in this regard falls on the shoulders of faculty members teaching in PharmD programs. Those faculty members should strike a fine balance between the supposed ideal roles of a PharmD graduate in a health care system, which is essential to advance the profession and push it forward, while helping PS develop realistic and practical expectations of their professional roles and opportunities to avoid professional disappointment upon experiencing real-life practice.

Most PS believed that PharmD jobs are well paid. PSs’ salary expectations are significantly higher compared to PP. This disparity should also be addressed and resolved through career awareness activities designed to inform the students about the career opportunities and their expected salaries and benefits [17].

### Factors that affect career choice of PharmD students and practitioners

General factors that may affect job selection among PS and PP were explored in this study. Both groups assigned the highest priority to the work environment. Interestingly, the second and third factors affecting job selection among the two groups differed significantly. PS were concerned with salary and benefits like bonus package and health insurance, while PP were more interested in the flexibility of the work schedule and number of days off. In comparison with students from other countries, Saudi PharmD students assigned the highest priority to moving up the job ladder and career development [25], PharmD students from USA indicated that opportunities to grow professionally in their desired setting to be more important than job-specific features, such as salary and benefits [26], and students from South Africa reported that service to community and paying back their sponsor as their main reasons for government hospital preference [27].

More than two-thirds of PS and PP agree on the ideal roles of PharmD practitioners as defined by the ACCP [18]. However, significant differences between PharmD students and practitioners in their expectations of the ideal role of PharmD graduates were noticed. Interestingly, almost 80% of PP believe that PharmD graduates have prescribing responsibility and should be able to provide repeat medication independently while only 49.1% PharmD students agree with that. Independent prescribing of medications is one of the evolving roles of PharmD practitioners and pharmacists around the world. Several countries have granted pharmacists the right of independent prescribing [28–31], and the improved outcomes in medication utilization, as well as clinical and cost outcomes in both inpatient hospital and ambulatory clinic (e.g., stroke, cancer pain) settings were clearly indicated [32–34].In Jordan, pharmacists are not granted the right of independent prescribing by regulatory authorities which may explain the low percentage of students perceiving independent prescribing as one of their ideal roles and missions.

Another important difference in the compounding procedures of medicines like IV antibiotics and TPN was also detected. This can be attributed to gaps in the university curricula as PS are not thoroughly exposed to compounding procedures in both theoretical and practical courses in their years of study. Furthermore, absence of ideal mentorship in undergraduate courses represents an important factor as most faculty members at the schools of pharmacy do not practice in a clinical setting creating a chasm between real-world needs and challenges and the education and training provided to the students [35].

Curricula of the PharmD programs in Jordan should be revisited and restructured to involve more theoretical and practical courses that introduces PS to compounding procedures carried out in clinical practice. In addition, virtual and simulation programs to train students in the necessary pharmacy-related skills are strongly recommended.

### Ideal vs current role of PharmD graduate from students’ and practitioners’ point of view

The most alarming outcomes are seen upon comparing the ideal and current role of PharmD practitioners in practice setting. Many of the ideal roles are currently practiced by less than 50% of PP despite the fact that some of these roles are not limited to a hospital setting and can be practiced in community pharmacies where most PP actually work. Updating clinical records, conducting basic physical assessments, reviewing immunizations and providing vaccines when required, designing prevention, intervention, and educational strategies for individuals and communities to manage chronic diseases and improve health and wellness are just few examples of such roles.

On the other hand, many of the ideal roles are not supported by the local regulations in Jordan or not acknowledged by insurance companies. For example, PP are neither authorized to independently prescribe medications as discussed earlier nor to order lab tests. Furthermore, clinics managed by PP do not exist in Jordan.

Advancements and changes to the legislations that govern the health care system should be employed to ensure that PP can perform their normal roles and missions.

As expected, more than two-thirds of PharmD roles that are currently practiced are those related to drug information and education. These include answering medication-related questions from patients and health care professionals, counseling and education on medication management, providing evidence-based drug information and recommendations, providing non-pharmacological treatments as well as educating individuals and communities to manage chronic diseases and improve health and wellness. The high rate of practice of such roles can be partially explained by the fact that many PP are considered drug experts, and many of these roles can be easily practiced in any practice setting. These roles are usually emphasized and appreciated by other health care providers including nurses, physicians and general practitioners around the world and in Jordan [1, 36–39]. For example, a study by Tahaineh et al. 2019 concluded that physicians' perceptions, expectations, and experiences towards the professional role of pharmacists have changed to become more positive over a period of 10 years before the study [40].

## Conclusion

It is evident that there are disparities between both the job expectations and perceived roles by students and those exhibited by practitioners after exposure to the job market and having practical experience. It is also significant that working in community pharmacies is not highly preferred by students despite the fact that it represents a significant proportion of the available job vacancies. Efforts must be put by schools in (i) modifying curricula to emphasize the compounding responsibilities of pharmacists and (ii) emphasize the clinical professional experience requirements for academics so as to be able to act as professional role models in addition to teachers; (iii) collaborate with professional organizations to work on the legal and regulatory frameworks necessary for PharmD graduates to assume their roles including prescription privileges in suitable professional setting; and (iv) increase exposure of students to community pharmacy and public health aspects of the professional practice of pharmacy.

## Data Availability

The datasets used and analyzed during the current study are available from the corresponding author on reasonable request.

## Abbreviations

ACCP: American College of Clinical Pharmacy
BSc Pharm: Bachelor of Science in Pharmacy
GPA: Grade point average
IV: Intravenous
JUST: Jordan University of Science and Technology
NA: Not applicable
PharmD: Doctor of Pharmacy
PP: PharmD practitioners
PS: PharmD students
QIP: Quality improvement plan
TPN: Total parenteral nutrition
UJ: University of Jordan
